# An Unusual Case of *Brucella abortus* Prosthetic Joint Infection

**DOI:** 10.7150/jbji.37096

**Published:** 2019-11-06

**Authors:** Jennifer Walsh, Anne Gilleece, Lynda Fenelon, David Cogley, Kirsten Schaffer

**Affiliations:** 1 St Vincent's University Hospital, School of Medicine, University College Dublin, Dublin 4, Ireland.; 2 Hermitage Medical Clinic, Old Lucan Road, Dublin 20, Ireland.; 3 Midlands Regional Hospital, Tullamore, Co Offaly, Ireland.

## Abstract

Brucellosis is a systemic infection caused by *brucella* species. Prosthetic joint infection due to *brucella* species is rare. We report the case of a prosthetic joint infection presenting fourteen years post treatment for systemic brucellosis.

A 75 year old cattle farmer underwent an uneventful left sided cemented total hip replacement for degenerative disease. Six months later his cattle herd was slaughtered due to the detection of brucellosis. He was admitted to hospital one month later for investigation of fevers and generalised fatigue. He reported drinking unpasteurised milk. He was diagnosed with brucellosis based on serological results, treated with intravenous gentamicin followed by oral doxycycline and symptoms resolved.

Six months later he reported no further systemic symptoms but noted occasional hip pain and on examination there was tenderness over the greater trochanter. An x-ray of the hip showed the prosthesis was in a satisfactory position but there was some loosening of the acetabular component. CRP was 3 and ESR was 18. Four months later a sinus developed over the greater trochanter. He was admitted to hospital for exploratory surgery. At the time of surgery the sinus was excised and the trochanteric clip was removed as it was loose. The wires were cut as close as possible to the trochanter. The remaining wires were embedded in cement and not removed. The prosthesis remained in situ. Intraoperative samples grew *Staphylococcus epidermidis*. He was treated with two weeks of intravenous flucloxacillin. The patient declined two stage revision surgery and was discharged on suppressive therapy with oral flucloxacillin and fusidic acid. On review six weeks post operatively he was feeling well, the wound had healed well, x-ray confirmed good positioning and there was no concern regarding ongoing infection. One month later fusidic acid was stopped. He was then reviewed annually for assessment of symptoms and monitoring of inflammatory markers and remained on oral flucloxacillin.

Thirteen years later he reported left sided hip pain. Radiographs showed loosening and cavitary defect of the acetabular component of the cemented hip replacement. He agreed to two stage reconstructive surgery. Three of six tissue samples from the first stage of reconstructive surgery grew a Gram negative coccobacillus on enrichment culture. The VITEK® 2 identified this isolate as *Brucella melitensis* (99%). Specific culture media for detection of *Brucella* had not been used. The sample was sent to the *Brucella* reference unit at the Animal and Plant Health Agency Liverpool and the isolate was confirmed as *Brucella abortus*. The patient completed two weeks of intravenous gentamicin (5mg/kg once daily) and three months of oral doxycycline (100mg twice daily) and rifampicin (450mg twice daily). Tissue specimens from the second stage of reconstructive surgery were negative. He is currently symptom free.

Following the detection of *Brucella* laboratory staff deemed high risk and theatre staff present at the time of the first stage of reconstructive surgery were advised to take post exposure prophylaxis. Post exposure prophylaxis with oral doxycycline 100mg twice daily for 21 days was recommended as per Public Health England recommendations. As follow up of exposed individuals was arranged via the occupational health department we are unable to comment on follow up serology samples. We are unaware of any cases of treatment failure. For the second stage of surgery operating staff wore orthopaedic space suits and used N95 face masks. Only essential operating room personnel were present.

Brucellosis is a systemic infection caused by *Brucella* species. Brucellosis is one of the most frequently encountered zoonotic diseases worldwide.^1^ Four species cause most cases of human disease, each with a different animal host reservoir: *Brucella melitensis* (goats, camels) is most common, followed by *Brucella abortus* (cattle), *Brucella suis* (pigs) and *Brucella canis* (dogs).^2^ Brucellosis is now a rare infection in humans in the EU, however, it is a severe disease with most infected cases hospitalised and with one death reported in 2016.^3^

Periprosthetic joint infection (PJI) due to *Brucella* species is rare. 30 cases have been documented worldwide to date. Time interval between implantation of prosthesis and diagnosis of PJI in cases documented to date ranges from immediately post operatively to 168 months with a median time interval of 48 months.^4^ Time between implantation and diagnosis of infection in this case was 180 months and is the longest interval to date to our knowledge. Most documented cases of *Brucella* prosthetic joint infection described are *Brucella* melitensis i.e. 23 of 30 cases. Regarding risk factors for *Brucella* infection information is available for 23 cases. 12 patients had consumed unpasteurised dairy products, 6 patients worked as a farmer or shepherd and 5 patients reported contact with cattle or goats.^4^The optimal surgical approach to PJI is unknown because of the lack of large-scale, randomized, prospective studies. Successful outcomes have been reported following one and two stage exchange. Patients without implant loosening have been treated conservatively with good outcomes. Antimicrobial treatment consists of doxycycline and rifampicin with or without an aminoglycoside. Duration of antimicrobial therapy has ranged from 6 weeks to 2 years in cases documented to date with a median of 16 weeks.^4^

This case highlights a number of teaching points. The patient was treated for brucellosis and systemic symptoms resolved. When the patient presented with pain and tenderness at the prosthetic joint site *Brucella* infection was not suspected, as a result precautions were not taken during his initial surgery and specific culture media for *Brucella* was not used. It is possible that *Brucella* was present at that time and not detected. Isolation of *Brucella* during the first stage of two stage revision surgery was fortuitous as the Vitek®2 identified the gram negative coccobacillus as *Brucella melitensis* and specific culture media for *Brucella* had not been used*.* Precautions were not taken during the first stage procedure and therefore theatre and laboratory staff were potentially exposed. This highlights the importance of thorough history taking in all patients presenting with possible prosthetic joint infection. A past history of brucellosis is a risk factor for chronic infection or relapsed infection which may affect any prosthesis and must be considered to ensure an accurate diagnosis is made and appropriate precautions are taken during all procedures. Previous authors have reported drinking unpasteurised milk and exposure to animals such as cattle, sheep and goats as risk factors for brucellosis and *Brucella* prosthetic joint infection.^4^ Patients with a prosthesis in situ should be alerted to the need to avoid such risk factors.

This case may represent reinfection as the patient continued to work as a farmer, chronic asymptomatic infection or relapsed infection. It is possible the patient did not have a sufficient treatment course for initial management of brucellosis and presented with a probable relapse following haematogenous seeding to the joint. While the VITEK®2 fortuitously identified the organism as* Brucella* it was identified as *Brucella melitensis* and subsequent identification at reference laboratory level was *Brucella abortus* and therefore one must be aware of obtaining VITEK®2 results which may not be 100% specific.

Brucellosis is now a rare infection. 30 cases of periprosthetic joint infection have been documented to date. To our knowledge this case represents the longest documented interval between implantation of device and development of infection. In addition, the patient was treated for brucellosis, systemic symptoms resolved, and *Brucella* PJI was diagnosed fourteen years following initial infection. This highlights the need for accurate history taking to facilitate appropriate investigation, management and infection control precautions.
